# Clinical, Laboratory, and Radiological Predictors of Three-Month Outcome After Surgical Treatment of Acute Extra-Axial Hematomas

**DOI:** 10.3390/jcm15155797

**Published:** 2026-07-24

**Authors:** Bartłomiej Kulesza, Mateusz Krakowiak, Agnieszka Brzozowska, Cezary Grochowski, Ryszard Maciejewski

**Affiliations:** 1 Institute of Medical Sciences, Faculty of Medicine, John Paul II Catholic University of Lublin, Racławickie Avenue 14, 20-950 Lublin, Poland; 2 Department of Neurosurgery, Stefan Cardinal Wyszyński Provincial Specialist Hospital Independent Public Health Care Centre, Kraśnicka 100, 20-718 Lublin, Poland; 3 Faculty of Medical Sciences and Health Sciences, Radom University, 29 Malczewskiego Street, 26-600 Radom, Poland; 4 Department of Information Technology and Medical Statistics with E-Health Laboratory, Faculty of Medical Sciences, Medical University of Lublin, 1 Racławickie Avenue, 20-059 Lublin, Poland

**Keywords:** traumatic brain injury, acute subdural hematoma, epidural hematoma, functional outcome

## Abstract

**Background:** Prognostic assessment in patients undergoing surgical treatment for acute extra-axial hematomas remains challenging. Most available prognostic models have been developed in heterogeneous populations of patients with traumatic brain injury, while relatively few studies have focused exclusively on surgically treated patients with acute subdural hematoma (ASDH) and epidural hematoma (EDH). Although mortality has been widely investigated, less attention has been paid to early functional recovery after surgery. This study aimed to identify clinical, physiological, laboratory, and radiological factors associated with early functional outcome three months after surgery in this patient population. **Methods:** We retrospectively analyzed 181 consecutive patients who underwent surgical treatment for acute extra-axial hematomas at two neurosurgical centers. Demographic, physiological, laboratory, and radiological variables recorded on admission were evaluated. Functional outcome was assessed three months after surgery using the Glasgow Outcome Scale (GOS). Favorable outcome was defined as GOS 4–5 and unfavorable outcome as GOS 1–3. Independent prognostic factors were identified using multivariable logistic regression analysis. **Results:** An unfavorable outcome was observed in 105 patients (58.0%). Multivariable analysis identified higher admission GCS score as the strongest independent predictor of a favorable functional 3-month outcome (OR = 1.45, 95% CI: 1.27–1.65; *p* < 0.001). The presence of an epidural hematoma (OR = 5.77, 95% CI: 1.36–24.47; *p* = 0.018) was also independently associated with a favorable functional outcome. Conversely, traumatic subarachnoid hemorrhage (OR = 0.19, 95% CI: 0.07–0.52; *p* = 0.001), hyperglycemia (OR = 0.20, 95% CI: 0.05–0.78; *p* = 0.020), and low hemoglobin levels (reference: normal hemoglobin; OR = 0.18, 95% CI: 0.06–0.54; *p* = 0.002) were associated with a lower likelihood of favorable functional outcome. **Conclusions:** Admission neurological status, represented by the GCS score, was the strongest predictor of 3-month functional outcome in patients undergoing surgical treatment for acute extra-axial hematomas. Hematoma type, hemoglobin level, traumatic subarachnoid hemorrhage, and hyperglycemia were also independently associated with outcome. These findings may improve early prognostic assessment; however, because of the retrospective study design and the lack of external validation, they should be interpreted with caution.

## 1. Introduction

Traumatic brain injury (TBI) remains a major cause of mortality and long-term disability worldwide, placing a significant burden on healthcare systems [1,2]. Among the most severe consequences of TBI are intracranial hematomas, which frequently necessitate urgent neurosurgical treatment. Acute subdural hematoma (ASDH) and epidural hematoma (EDH) are the most common forms of traumatic extra-axial hematomas [3].

Despite advances in neurosurgical and neurocritical care, mortality among patients undergoing surgical treatment for acute subdural hematoma remains high, reaching up to 60% in some series. Acute epidural hematoma is generally associated with a more favorable prognosis [4,5]. Furthermore, many survivors experience substantial long-term neurological disability, often requiring assistance with basic activities of daily living. Following discharge, a considerable proportion of patients require ongoing care in rehabilitation units or long-term healthcare facilities rather than returning directly to their homes [6]. In addition to its clinical and socioeconomic consequences, traumatic brain injury places a considerable psychological and emotional burden on patients’ families, especially given that TBI predominantly affects young adults during their most productive years [7].

In recent years, numerous studies have investigated prognostic factors in broad populations of patients with traumatic brain injury (TBI). However, relatively few studies have focused specifically on patients requiring surgical treatment for acute subdural hematoma (ASDH) or epidural hematoma (EDH). Moreover, most available prognostic models have been developed using heterogeneous TBI populations, which may limit their applicability to patients with extra-axial hematomas. While early mortality remains an important endpoint after traumatic brain injury, functional recovery among survivors is equally important because it reflects the patient’s level of independence and long-term neurological status. Therefore, the aim of the present study was to identify clinical, laboratory, and radiological factors associated with early functional outcome three months after surgical treatment of acute extra-axial hematomas. A strength of the present study is the assessment of prognostic factors in relation to three-month outcome, whereas most previous studies have focused either on in-hospital mortality or on long-term functional outcomes assessed six months after injury. Three-month outcome was selected because it reflects early functional recovery after surgical treatment and is commonly used to evaluate neurological recovery during the early post-injury period. To our knowledge, few studies have comprehensively evaluated clinical, physiological, laboratory, and radiological predictors of 3-month functional outcome in surgically treated patients with acute extra-axial hematomas.

## 2. Materials and Methods

### 2.1. Patients and Study Design

**Figure 1:**
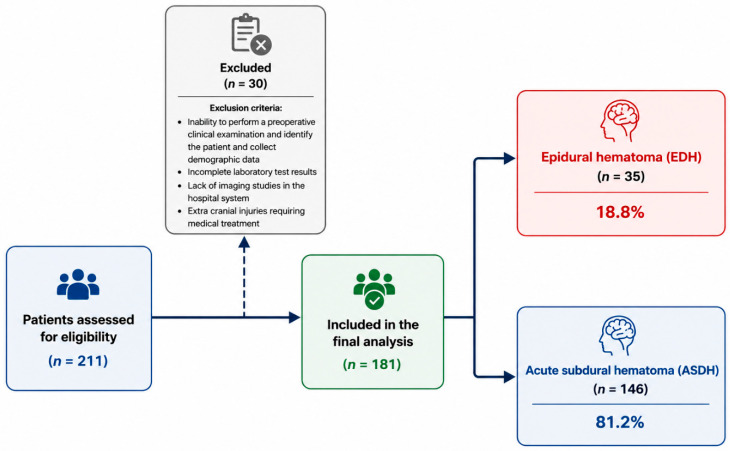
Flowchart of patient selection. Arrows indicate the division of the study population into the epidural hematoma and acute subdural hematoma groups.

At the Department of Neurosurgery of the University Clinical Hospital No. 4 in Lublin, 119 patients underwent surgical treatment between 1 January 2019 and 30 October 2020. An additional 92 patients underwent surgical treatment at the Department of Neurosurgery of the Regional Specialist Hospital in Lublin between 1 February 2024 and 15 February 2026. After applying the exclusion criteria, including incomplete medical records, unavailable imaging studies, the presence of extracranial injuries requiring treatment, and a lack of clinical assessment at three-month outcome, a total of 181 patients were included in the final analysis (Figure 1).

Clinical, laboratory, and radiological parameters were retrospectively extracted from the medical records of all enrolled patients. The analyzed variables were categorized into four groups: demographic, clinical, laboratory, and imaging parameters.

Demographic data included age and sex. Clinical parameters assessed at admission comprised the Glasgow Coma Scale (GCS) score, pupillary reactivity, systolic blood pressure (SBP), heart rate (HR), respiratory rate (RR), and peripheral oxygen saturation (SpO_2_). Respiratory rate was considered abnormal when it was below 10 or above 25 breaths per minute. Oxygen saturation values below 96% were considered abnormal. Systolic blood pressure was considered abnormal when it was below 90 mmHg or above 140 mmHg.

The admission GCS score reflected the initial neurological assessment documented in the medical records and was not adjusted for blood alcohol concentration.

Laboratory evaluation included white blood cell count (WBC), hemoglobin concentration (HGB), platelet count (PLT), neutrophil-to-lymphocyte ratio (NLR), platelet-to-lymphocyte ratio (PLR), serum glucose level, sodium and potassium concentrations, coagulation abnormalities, and blood alcohol concentration. Coagulopathy was defined as the presence of at least one of the following abnormalities: international normalized ratio (INR) > 1.5, activated partial thromboplastin time (APTT) > 35 s, or platelet count < 100 × 10^3^/µL. Hyperglycemia was defined as a blood glucose level > 160 mg/dL. An NLR value > 5 was considered abnormal. Low hemoglobin was defined as hemoglobin < 12 g/dL.

All patients underwent non-contrast head computed tomography (CT) on admission. Radiological assessment included the presence of skull fractures, traumatic subarachnoid hemorrhage (tSAH), intraventricular hemorrhage (IVH), cerebral contusions, hematoma thickness, midline shift (MLS), and basal cistern status. Measurements of hematoma thickness and MLS were performed on preoperative CT scans using OsiriX MD version 11.0.4 image analysis software.

Outcome was assessed three months after surgery using the Glasgow Outcome Scale (GOS). For patients who were no longer hospitalized, follow-up information was obtained through a telephone interview with the patient or a family member. Based on the collected information, patients were classified according to the GOS. A favorable outcome was defined as a GOS score of 4–5, whereas an unfavorable outcome was defined as a GOS score of 1–3.

### 2.2. Statistical Analysis

The obtained data were subjected to statistical analysis. Continuous variables were presented as means, medians, quartiles, and standard deviations, whereas categorical variables were expressed as counts and percentages.

The normality of distribution for continuous variables was assessed using the Shapiro–Wilk test. Categorical variables were compared using Pearson’s chi-square test. Variables associated with functional outcome in the univariate analysis (*p* < 0.05) were considered as candidate predictors for the multivariable logistic regression model. Admission GCS was entered into the multivariable model as a continuous variable instead of the dichotomous severe TBI variable used in the univariate analysis in order to preserve prognostic information. Because epidural hematoma (EDH) and acute subdural hematoma (ASDH) were mutually exclusive categories, only EDH was included in the multivariable model, with ASDH serving as the reference category. Abnormal respiratory rate was not entered into the model because complete separation prevented reliable estimation of regression coefficients using standard logistic regression. A backward stepwise selection procedure was applied, with variables showing *p* > 0.05 sequentially removed until the final model was obtained. Favorable outcome (GOS 4–5) was defined as the event of interest. Complete-case analysis was performed because patients with incomplete data had been excluded before statistical analysis. Multicollinearity among the candidate variables was assessed before model construction, and no clinically relevant collinearity was identified.

A *p*-value of less than 0.05 was considered statistically significant. Statistical analyses were performed using STATISTICA 13.0 software (StatSoft, Kraków, Poland).

## 3. Results

### 3.1. Patient Characteristics

A total of 181 patients who underwent surgical treatment for acute extra-axial hematomas were included in the study. The mean age of the study population was 56.3 ± 19.8 years, and males accounted for 82.9% of all patients. Acute subdural hematoma was the lesion diagnosed in 80.6% of cases, whereas epidural hematoma was present in 19.3% of patients.

Clinical and physiological parameters of the study population are presented in Table 1. The median admission Glasgow Coma Scale score was 7 points, and severe traumatic brain injury (GCS ≤ 8) was observed in more than half of the patients. Abnormal pupillary response was present in 40.3% of cases. The median systolic blood pressure was 140 mmHg, median oxygen saturation was 97%, and median respiratory rate was 16 breaths per minute.

Baseline laboratory findings are summarized in Table 1. Leukocytosis was the most common laboratory abnormality, followed by abnormal platelet count and elevated neutrophil-to-lymphocyte ratio. Hyperglycemia and coagulopathy were observed in 16.0% and 14.4% of patients, respectively.

Radiological parameters are presented in Table 2. Midline shift was the most frequent radiological abnormality and was identified in 82.9% of patients, with a median displacement of 10 mm. Traumatic subarachnoid hemorrhage was present in 40.3% of cases, while intraventricular hemorrhage occurred in 15.5% of patients. Compressed and obliterated basal cisterns were observed in 29.8% and 22.7% of patients, respectively.

Treatment outcomes are summarized in Table 3. An unfavorable outcome (GOS 1–3) was observed in 58.0% of patients, whereas a favorable outcome (GOS 4–5) was achieved in 42.0%.

Functional outcome was assessed three months after surgery using the Glasgow Outcome Scale (GOS). Favorable outcome was defined as GOS 4–5, whereas unfavorable outcome was defined as GOS 1–3.

### 3.2. Univariate Analysis

Univariate analysis demonstrated that unfavorable outcome occurred significantly more frequently in patients with severe neurological impairment on admission, abnormal respiratory rate, and reduced oxygen saturation (Table 4). Among laboratory variables, coagulopathy, hyperglycemia, and an elevated neutrophil-to-lymphocyte ratio (NLR) were significantly associated with unfavorable outcome, whereas leukocytosis and abnormal prothrombin time were not. Among radiological variables, intraventricular hemorrhage showed the strongest association with unfavorable outcome, affecting 92.9% of patients (*p* < 0.0001). Traumatic subarachnoid hemorrhage, absent basal cisterns, and acute subdural hematoma were also significantly associated with unfavorable outcome, whereas epidural hematoma was more frequently associated with a favorable outcome. No significant associations were observed for skull fractures, cerebral contusions, midline shift, compressed basal cisterns, or intracerebral hematoma (Table 4).

### 3.3. Multivariate Analysis

To identify independent factors associated with a favorable outcome, a multivariable logistic regression analysis was performed. Variables associated with functional outcome in the univariate analysis were considered as candidate predictors for the initial multivariable model. All eligible variables identified in the univariate analysis, except abnormal respiratory rate, were entered into the initial model. Admission GCS was analyzed as a continuous variable instead of the dichotomous severe TBI variable, EDH was used to represent hematoma type with ASDH serving as the reference category, and abnormal respiratory rate was excluded because complete separation prevented reliable estimation of regression coefficients. A backward stepwise selection procedure was then applied, resulting in the final model presented in Table 5.

The final multivariable logistic regression model was statistically significant (χ^2^ = 108.96, *p* < 0.0001). Higher admission GCS score and the presence of an epidural hematoma were independently associated with a favorable 3-month functional outcome. In contrast, low hemoglobin level, traumatic subarachnoid hemorrhage, and hyperglycemia were independently associated with a lower likelihood of favorable functional outcome.

Abnormal pupillary response, abnormal oxygen saturation, blood alcohol concentration, elevated neutrophil-to-lymphocyte ratio, coagulopathy, intraventricular hemorrhage, and obliterated basal cisterns were included in the initial multivariable model but were removed during backward stepwise selection because they did not remain independently associated with outcome and therefore were not retained in the final model. Detailed results are presented in Table 5.

## 4. Discussion

### 4.1. Demographic Characteristics

Demographic characteristics were not independently associated with three-month functional outcome in the present cohort. Nevertheless, increasing age has frequently been associated with poorer neurological recovery after traumatic brain injury [8,9]. A meta-analysis by Ma et al., including more than 2.2 million elderly patients with TBI, confirmed the unfavorable influence of increasing age on clinical outcome [10].

Sex was not associated with treatment outcome. Similar findings have been reported in studies of both heterogeneous traumatic brain injury populations and patients undergoing surgical treatment for epidural and acute subdural hematomas, where sex was not identified as an independent predictor of functional outcome [3,11,12,13]. In the present cohort, neurological status and injury-related factors appeared to be more relevant to early functional recovery than age or sex.

### 4.2. Physiological Parameters

Admission neurological status, reflected by the Glasgow Coma Scale, was the strongest factor associated with favorable functional recovery in the present cohort [14]. Previous studies have similarly shown that admission GCS score and pupillary reactivity are closely related to subsequent neurological outcome after TBI [14,15]. The relationship between admission GCS score and functional recovery appears to be nearly linear, with progressively lower scores associated with a greater likelihood of unfavorable outcome [15,16]. In our cohort, each one-point increase in the GCS score was associated with an approximately 45% higher likelihood of favorable functional recovery. This finding emphasizes the importance of the initial neurological examination when estimating the potential for recovery during the first three months after surgery.

Early physiological disturbances after traumatic brain injury may contribute to secondary brain injury and limit subsequent neurological recovery. Autonomic nervous system dysfunction is common after TBI and may result in abnormalities of blood pressure, heart rate, and respiratory function. Consequently, impaired systemic physiology may compromise cerebral perfusion and oxygen delivery, reducing the potential for favorable functional improvement [2,17]. Hypoxia and hypotension are among the most important secondary insults that may adversely affect neurological recovery following traumatic brain injury [17,18]. Kalayci et al., in a cohort consisting exclusively of patients with acute subdural hematoma, demonstrated that oxygen saturation ≤ 96% on admission was significantly associated with an unfavorable outcome [19]. This finding is consistent with the results of our univariate analysis, in which oxygen saturation ≤ 96% was also significantly associated with an unfavorable functional outcome. Previous studies have demonstrated that a respiratory rate outside the normal range (10–25 breaths/min) and a heart rate outside the range of 70–120 beats/min are associated with unfavorable outcome [14,19,20]. This observation is partially supported by the findings of our study. In addition to conventional physiological assessment, multimodal neuromonitoring is becoming increasingly important in patients with severe traumatic brain injury. Forcione et al. showed that brain tissue oxygen tension (PbtO_2_) and near-infrared spectroscopy (NIRS) often provide different information about cerebral oxygenation. They concluded that no single monitoring method is sufficient to fully assess cerebral oxygenation and metabolism and recommended combining ICP, CPP, PbtO_2_, NIRS, and other neuromonitoring techniques. Although multimodal neuromonitoring was not used in our study, these techniques may complement routine admission assessment and help identify patients at risk of limited neurological recovery [21].

### 4.3. Laboratory Parameters

Routine laboratory parameters measured on admission may reflect systemic disturbances that influence neurological recovery after traumatic brain injury. Among these variables, hyperglycemia and low hemoglobin level remained independently associated with three-month functional outcome. Elevated blood glucose levels commonly occur immediately after traumatic brain injury as part of the systemic stress response and may contribute to secondary brain injury by promoting oxidative stress, disrupting the blood–brain barrier, enhancing the inflammatory response, and exacerbating cerebral edema [22,23,24]. Consistent with previous reports, admission hyperglycemia was independently associated with a lower likelihood of achieving favorable functional recovery at three months. This finding suggests that hyperglycemia may reflect not only injury severity but also biological processes that impair subsequent neurological improvement. Coagulopathy was associated with functional outcome in the univariate analysis but was not retained in the final multivariable model. Previous studies have shown that trauma-induced coagulopathy is associated with unfavorable outcome after severe traumatic brain injury [25,26,27].

Systemic inflammatory markers may reflect the intensity of the post-traumatic inflammatory response and its potential influence on neurological recovery [28,29]. Among these markers, the neutrophil-to-lymphocyte ratio has been reported to provide more consistent prognostic information than leukocyte count alone. Elevated NLR has been consistently associated with unfavorable outcomes in patients with traumatic brain injury [30,31]. A related but less established inflammatory marker is the platelet-to-lymphocyte ratio, which has also been reported to have prognostic value [32]. In our study, elevated NLR was associated with unfavorable functional outcome in the univariate analysis, but this relationship was no longer independent after adjustment for other admission variables.

Adequate hemoglobin concentration is important for maintaining cerebral oxygen delivery during the early phase of recovery after traumatic brain injury. Reduced oxygen-carrying capacity may compromise vulnerable brain tissue and limit subsequent neurological improvement. Consistent with previous reports [33,34], low hemoglobin level was independently associated with a lower likelihood of favorable functional outcome at three months in our cohort.

Previous studies have demonstrated that both hyponatremia and hypernatremia are associated with an increased risk of unfavorable functional outcomes [35,36]. However, our findings did not confirm this association.

Alcohol intoxication is common among patients with traumatic brain injury [37]. Earlier studies suggested that ethanol might exert a neuroprotective effect following traumatic brain injury [38]. However, subsequent studies failed to confirm these findings, and current evidence does not support a beneficial effect of alcohol on neurological outcome [39,40]. Blood alcohol concentration above 0.2‰ was associated with functional outcome in the univariate analysis but was not retained as an independent predictor in the multivariable model. Therefore, this finding should be interpreted with caution.

### 4.4. Radiological Parameters

Radiological findings provide important information regarding the extent of primary brain injury and the potential for subsequent neurological recovery [41]. Greater midline shift and increased hematoma thickness have previously been associated with unfavorable neurological outcome [15,42,43]. However, neither variable was significantly related to three-month functional recovery in the present cohort. Traumatic subarachnoid hemorrhage and obliterated basal cisterns may indicate a more extensive intracranial injury and have been associated with unfavorable neurological outcome [9,15,44,45]. In our study, both findings were associated with unfavorable outcome in the univariate analysis; however, only tSAH remained independently associated with a lower likelihood of favorable functional recovery. Another radiological parameter associated with prognosis is intraventricular hemorrhage. Previous studies have shown that IVH is associated with increased mortality and poorer neurological outcomes [46,47]. Similarly, IVH was more frequently observed among patients who failed to achieve favorable functional recovery at three months. One of the most important prognostic factors is the type of extra-axial hematoma. Patients with acute subdural hematomas generally achieve poorer neurological recovery than those with epidural hematomas [3,48], which is consistent with our finding that EDH was independently associated with favorable three-month outcome. This difference likely reflects the more frequent coexistence of diffuse parenchymal injury and secondary intracranial lesions in patients with ASDH.

Overall, three-month functional recovery after surgical treatment of acute extra-axial hematomas was associated with a combination of neurological, systemic, and radiological factors. Admission GCS remained the dominant factor, whereas hyperglycemia, low hemoglobin level, tSAH, and hematoma type provided additional information regarding the likelihood of achieving favorable functional outcome. These findings complement studies focused on short-term mortality by emphasizing that survival and subsequent neurological recovery are not equivalent endpoints. Nevertheless, the observed associations remain exploratory and require confirmation in prospective multicenter studies with external validation.

## 5. Limitations

This study has several limitations. First, its retrospective design may have introduced selection and information bias, and residual confounding due to unmeasured variables cannot be excluded. In addition, perioperative variables, such as the time from injury to surgical evacuation, the surgical technique used (e.g., craniotomy versus decompressive craniectomy), and intraoperative findings were not available for all patients and therefore were not included in the analysis. These factors may also influence neurological outcome after traumatic brain injury. Despite the relatively large overall cohort, only 35 patients had epidural hematomas, which may have limited the statistical power of some subgroup analyses. In addition, the predominance of patients with acute subdural hematoma in the study cohort likely influenced the overall rates of unfavorable outcome and mortality, as ASDH is inherently associated with a substantially worse prognosis than EDH. Therefore, the overall outcome of the cohort should be interpreted in the context of the different natural history of these two extra-axial hematomas. Because outcome assessment for discharged patients was performed by telephone interview, some degree of outcome misclassification cannot be excluded. Furthermore, outcome assessment was based on the standard Glasgow Outcome Scale rather than the structured Glasgow Outcome Scale–Extended (GOSE) interview, which may have further contributed to outcome misclassification. In addition, the inclusion of patients from two neurosurgical centers may have introduced center-related differences in clinical management that were not adjusted for in the statistical analysis. Because recruitment center and calendar period were closely linked, their independent effects could not be separated and therefore were not adjusted for in the multivariable analysis. Furthermore, patients were recruited during two different time periods, and potential temporal changes in clinical practice, perioperative management, and intensive care were not analyzed or adjusted for. We also did not compare the baseline characteristics of excluded and included patients; therefore, the possibility of attrition bias cannot be excluded. Additionally, the identified prognostic factors were not externally validated. Because the primary objective of this study was to identify independent prognostic factors rather than to develop or validate a clinical prediction model, formal assessment of model discrimination, calibration, and internal validation was beyond the scope of the present study. Future prospective studies should include these analyses to determine the clinical utility of the identified prognostic factors. Finally, the study population was predominantly composed of patients with severe traumatic brain injury, with a median admission GCS score of 7 and more than half of the cohort presenting with GCS ≤ 8. Therefore, the findings may not be generalizable to patients with mild or moderate traumatic brain injury or to the broader spectrum of surgically treated extra-axial hematomas.

## 6. Conclusions

The admission Glasgow Coma Scale score was the strongest predictor of favorable functional outcome at three months in patients undergoing surgical treatment for acute extra-axial hematomas. In addition, epidural hematoma, normal hemoglobin level, the absence of traumatic subarachnoid hemorrhage, and the absence of hyperglycemia were independently associated with a favorable functional outcome. These findings provide further insight into factors associated with early functional outcome after surgical treatment of acute extra-axial hematomas. However, given the retrospective design of the study and the lack of external validation, they should be interpreted with caution and confirmed in prospective multicenter studies.

## Figures and Tables

**Table 1:** Baseline clinical, physiological and laboratory parameters of the study population.

Variable	Value
**Demographic characteristics**	
Age, years	58 (42–72)
Male sex, *n* (%)	150 (82.9)
**Clinical and physiological characteristics**	
Admission GCS score	7 (4–12)
Severe TBI (GCS ≤ 8), *n* (%)	100 (55.3)
Abnormal pupillary response, *n* (%)	73 (40.3)
Systolic blood pressure (SBP), mmHg	140 (116–160)
Heart rate, beats/min	80 (69–95)
Oxygen saturation, %	97 (95–98)
Respiratory rate, breaths/min	16 (14–18)
Blood alcohol concentration > 0.2‰, *n* (%)	53 (29.3)
**Laboratory characteristics**	
Glucose, mg/dL	140 (116–175)
INR	1.05 (0.96–1.15)
APTT, s	26.0 (23.6–28.9)
White blood cell count (×10^3^/µL)	11.1 (8.7–14.8)
Neutrophil-to-lymphocyte ratio (NLR)	5.87 (3.10–8.10)
Hemoglobin, g/dL	13.7 (12.1–14.6)
Platelet count (×10^3^/µL)	192 (134–250)
Sodium, mmol/L	140 (138–143)
Potassium, mmol/L	3.9 (3.6–4.35)
Leukocytosis, *n* (%)	105 (58.0)
Elevated NLR (>5), *n* (%)	54 (29.8)
Hyperglycemia, *n* (%)	29 (16.0)
Coagulopathy, *n* (%)	26 (14.4)

Data are presented as median (interquartile range) or *n* (%).

**Table 2:** Baseline radiological characteristics of the study population.

Variable	*n* (%)/Value
**Hematoma type**	
Epidural hematoma (EDH)	35 (19.3)
Acute subdural hematoma (ASDH)	146 (80.7)
**Associated intracranial findings**	
Skull fracture	54 (29.8)
Traumatic subarachnoid hemorrhage (tSAH)	73 (40.3)
Intraventricular hemorrhage (IVH)	28 (15.5)
Intracerebral hematoma	21 (11.6)
Cerebral contusion	41 (22.7)
**Mass effect**	
Midline shift present	150 (82.9)
Compressed basal cisterns	54 (29.8)
Obliterated basal cisterns	41 (22.7)
Midline shift, mm	10 (6–15)

Data are presented as median (interquartile range) or *n* (%). The observed range for midline shift was 2–38 mm.

**Table 3:** Three-month functional outcome of the study population.

Glasgow Outcome Scale (GOS)	*n* (%)
Favorable outcome (GOS 4–5)	76 (42.0)
Unfavorable outcome (GOS 1–3)	105 (58.0)

**Table 4:** Univariate analysis of factors associated with 3-month functional outcome.

Variable	Favorable Outcome *n* (%)	Unfavorable Outcome *n* (%)	*p*-Value
**Clinical and physiological variables**			
Severe TBI (GCS ≤ 8)	15 (15.0)	85 (85.0)	<0.0001
Abnormal pupillary response	25 (34.3)	48 (65.7)	<0.0001
Abnormal respiratory rate	0 (0.0)	16 (100.0)	0.0004
Abnormal oxygen saturation	8 (19.5)	33 (80.5)	0.001
Blood alcohol concentration > 0.2‰	31 (58.5)	22 (41.5)	0.004
**Laboratory variables**			
Leukocytosis	44 (42.7)	59 (57.3)	0.82
Elevated NLR (>5)	14 (25.9)	40 (74.1)	0.004
Hyperglycemia	5 (17.2)	24 (82.8)	0.004
Coagulopathy	3 (11.5)	23 (88.5)	0.001
Abnormal prothrombin time	18 (40.9)	26 (59.1)	0.87
Low hemoglobin level	8 (19.0)	34 (81.0)	0.001
**Radiological variables**			
Epidural hematoma (EDH)	28 (80.0)	7 (20.0)	<0.0001
Acute subdural hematoma (ASDH)	51 (34.9)	95 (65.1)	<0.0001
Skull fracture	24 (44.4)	30 (55.6)	0.66
Traumatic subarachnoid hemorrhage	15 (20.5)	58 (79.5)	<0.0001
Intraventricular hemorrhage	2 (7.1)	26 (92.9)	<0.0001
Intracerebral hematoma	5 (23.8)	16 (76.2)	0.07
Cerebral contusion	18 (43.9)	23 (56.1)	0.78
Midline shift present	61 (40.7)	89 (59.3)	0.43
Obliterated basal cisterns	5 (12.2)	36 (87.8)	<0.0001
Compressed basal cisterns	27 (50.0)	27 (50.0)	0.15

*p*-values were calculated using Pearson’s chi-square test.

**Table 5:** Multivariable logistic regression analysis of factors independently associated with favorable 3-month functional outcome.

Independent Predictor	Adjusted OR	95% CI	*p*-Value
Admission GCS score (per 1-point increase)	**1.45**	1.27–1.65	<0.001
Epidural hematoma (EDH)	**5.77**	1.36–24.47	0.018
Low hemoglobin level	**0.18**	0.06–0.54	0.002
Traumatic subarachnoid hemorrhage (tSAH)	**0.19**	0.07–0.52	0.001
Hyperglycemia	**0.20**	0.05–0.78	0.020

Abbreviations: OR, odds ratio; CI, confidence interval; GCS, Glasgow Coma Scale. The dependent variable in the logistic regression model was favorable functional outcome (GOS 4–5).

## Data Availability

The data presented in this study are not publicly available due to privacy and ethical restrictions involving patient data. De-identified data may be made available by the corresponding author upon reasonable request and with permission from the Institutional Ethics Committee.
